# Investigation of the Molecular Mechanisms by Which Endothelin-3 Stimulates Preadipocyte Growth

**DOI:** 10.3389/fendo.2021.661828

**Published:** 2021-05-21

**Authors:** An-Ci Siao, Li-Jane Shih, Yen-Yue Lin, Yi-Wei Tsuei, Yow-Chii Kuo, Hui-Chen Ku, Chih-Ping Chuu, Po-Jen Hsiao, Yung-Hsi Kao

**Affiliations:** ^1^ Department of Life Sciences, National Central University, Taoyuan, Taiwan; ^2^ Medical Laboratory, Taoyuan Armed Forces General Hospital, Taoyuan, Taiwan; ^3^ Graduate Institute of Medical Science, National Defense Medical Center, Taipei, Taiwan; ^4^ Department of Emergency Medicine, Taoyuan Armed Forces General Hospital, Taoyuan City, Taiwan; ^5^ Department of Emergency Medicine, Tri-Service General Hospital, National Defense Medical Center, Taipei, Taiwan; ^6^ Department of Gastroenterology, Landseed Hospital, Taoyuan, Taiwan; ^7^ Department of Pediatrics, Taipei Tzu Chi Hospital, Buddhist Tzu Chi Medical Foundation, New Taipei City, Taiwan; ^8^ Institute of Cellular and System Medicine, National Health Research Institutes, Zhunan, Taiwan; ^9^ Division of Nephrology, Department of Internal Medicine, Taoyuan Armed Forces General Hospital, Taoyuan City, Taiwan; ^10^ Division of Nephrology, Department of Internal Medicine, Tri-Service General Hospital, National Defense Medical Center, Taipei, Taiwan

**Keywords:** endothelin-3, preadipocyte, AMP-activated protein kinase, signal transducer and activator of transcription, c-Jun

## Abstract

Endothelins induce many biological responses, and they are composed of three peptides: ET-1, ET-2, and ET-3. Reports have indicated that ET-1 regulates cell proliferation, adipogenesis, and other cell responses and that ET-3 stimulates the growth of gastrointestinal epithelial cells and melanocytes. However, the signalling pathways of ET3 that mediate the growth of fat cells are still unclear. Using 3T3-L1 white preadipocytes, we found that ET-3 induced increases in both cell number and BrdU incorporation. Pretreatment with an ET_A_R antagonist (but not an ET_B_R antagonist) blocked the ET-3-induced increases in both cell number and BrdU incorporation. Additionally, BQ610 suppressed the ET-3-induced increases in phosphorylation of AMPK, c-JUN, and STAT3 proteins, and pretreatment with specific inhibitors of AMPK, JNK/c-JUN, or JAK/STAT3 prevented the ET-3-induced increases in phosphorylation of AMPK, c-JUN, and STAT3, respectively. Neither p38 MAPK inhibitor nor PKC inhibitor altered the effects of ET-3 on cell growth. These data suggest that ET-3 stimulates preadipocyte growth through the ET_A_R, AMPK, JNK/c-JUN, and STAT3 pathways. Moreover, ET-3 did not alter HIB1B brown preadipocyte and D12 beige preadipocyte growth, suggesting a preadipocyte type-dependent effect. The results of this study may help explain how endothelin mediates fat cell activity and fat cell-associated diseases.

## Introduction

Obesity is one of the key risk factors leading to cardiovascular diseases, and its development is associated with an increase in the number of fat cells and the accumulation of triglycerides in fat cells (triglyceride accumulation due to mitogenesis and differentiation) (1). These processes can be regulated by endocrine factors, growth factors, and endothelins (ETs) (2–5). ETs, which were discovered by Yanagisawa in 1988, induce many biological responses, including cell proliferation and death (6). The ET family consists of three small (21 amino acid) peptides: ET-1, ET-2, and ET-3. ET-1 and ET-2 have similar structures, whereas ET-3 differs by 6 of 21 amino acids (6, 7). ET-1 has been reported to be closely associated with obesity and obesity-associated diabetes, hypertension and cardiovascular disease (1–3, 8); however, whether ET-3 alters fat cell activity and obesity-associated disease is unknown. Expression of ET-3 has been found in the brain (9) and spinal cord (10), where it is believed to regulate cellular growth and development. Other sources of ET-3 include glomerular epithelial cells, kidney medullary tissue, plasma, and cirrhotic hepatocytes (2). ET-3 has been detected in other tissues, including the heart (11), endometrium (12, 13), and pituitary. Interestingly, a previous report indicated that the pituitary has higher levels of ET-3 than ET-1 (11). In the intestinal epithelium, ET-3 stimulates epithelial cell secretion of ions. ETs are upregulated in acute and chronic intestinal inflammation as well as colon carcinoma to foster cell proliferation and survival (14–16).

ET-3 predominantly acts locally in a paracrine or autocrine fashion. In mammalian cells, ETs have many effects mediated by two different subtypes of G protein-coupled receptors: ET_A_ and ET_B_ (17, 18). These two receptors are widespread in the body; however, their expression levels vary with tissue type. Both ET_A_R and ET_B_R are found in the smooth muscle tissue of blood vessels, with ET_B_R primarily expressed in the endothelial cells that line the interior of the blood vessels (8) and ET_A_R primarily expressed in L6 myoblasts (19). Because both subtypes of ET receptors are also found in blood vessels and cells of the brain, choroid plexus and peripheral nerves, they may mediate neurotransmission and vascular functions (20). ET_A_R can increase retention of sodium and contraction of the blood vessels and leads to increased blood pressure (17), while ET_B_R causes nitric oxide release and results in lower blood pressure (18, 21). In fat cells, ET_A_R was expressed in isolated rat adipocytes and 3T3-L1 adipocytes (22). Northern blot analysis showed that 3T3-L1 adipocytes and rat primary adipocytes expressed abundant ET_A_R mRNA levels but did not express ET_B_R mRNA (23). This finding suggests that ET_A_R, but not ET_B_R, is the predominant endothelin receptor in rodent fat cells. An elevated serum ET-3 concentration is associated with a number of developmental and growth processes, such as neural crest-derived epidermal melanocytes and enteric neurons, natriuresis, and melanocyte growth (2, 7, 24, 25).

ET-3 is reported to regulate cellular actions through ET_B_R engagement, and subsequent phosphorylation of p42/44 results in enhanced transcription of the immediate early response genes *c-fos* and *c-jun*, a process commonly assumed to be mediated by ET_A_R, and increased cell growth and relative cell area (26). However, the mechanisms by which ET-3 affects the proliferation and cell growth of preadipocytes remain unknown. The ET family contains ET-1, ET-2, and ET-3, which have different distributions in tissues and distinct receptor binding affinities in nonfat cells and fat cells (2); thus, it is worthwhile to explore whether ET-1 and ET-3 have different effects on the growth of 3T3-L1 white preadipocytes, HIB1B brown preadipocytes, and D12 beige preadipocytes. In fat cells, ET-1 was found to regulate cell differentiation, glucose uptake, lipid metabolism, and adipokine secretion (25). Despite the importance of ET-1 on fat cells, whether ET-3 affects preadipocyte growth and its signalling pathways in fat cells are unknown. In the present study, we used 3T3-L1 preadipocytes to determine the influence of the various ET-3 signalling cascades on the growth of fat cells. We found that ET-3 stimulates preadipocyte growth through the ET_A_R, AMPK, JAK2/STAT3, and JNK/cJUN pathways.

## Materials and Methods

### Chemical Reagents

All materials (e.g., dimethyl sulfoxide (DMSO), penicillin, and streptomycin) were purchased from Sigma (St. Louis, MO, USA), and other culture reagents (e.g., Dulbecco’s modified Eagle’s medium (DMEM)) were obtained from Gibco BRL-Invitrogen (New York, NY, USA). Antibodies were purchased from Cell Signaling Technology (Danvers, MA, USA) or Santa Cruz Biotechnology (Santa Cruz, CA, USA) (**Supplementary Table 1**). Inhibitors were purchased from Bachem (Bubendorf, Switzerland), Enzo Life Sciences, Inc. (New York, NY, USA), Cayman Chemical (Ann Arbor, MI, USA), or Tocris Bioscience (Bristol, UK).

### Cell Culture

We obtained 3T3-L1 preadipocytes and D12 beige preadipocytes from ATCC (Manassas, VA, USA) and HIB1B preadipocytes from Professor Heng Lin (Taipei Medical University, Taiwan). Cells were grown according to the method described by Siao et al. (27) in DMEM containing 10% CS (3T3-L1 cells), 10% FBS (HIB1B cells), 15% FBS (D12 cells), 100 units/ml penicillin, and 100 µg/ml streptomycin in a humidified atmosphere of 95% air and 5% CO_2_ at 37°C. The culture medium was replaced every two days.

### Growth Stimulation Experiments

All cells were seeded at 2 x 10^4^ cells/well in a 12-well culture plate. After 24 h, the cells were treated with or without ET-3 at various concentrations (0-100 nM). After 48 h of incubation, the cells were stained with 0.4% trypan blue and counted on a hemocytometer. Cell proliferation was detected by a commercial bromodeoxyuridine (BrdU) ELISA kit (Roche Applied Science; Mannheim, Germany) (27). Briefly, 2,000 cells/well were seeded in a 96-well microplate at 37°C. After allowing 24 h for attachment, cells were starved with serum-free DMEM for 36 h. The medium was then replaced with fresh DMEM containing ET-3 for 18 h at 37°C. After treatment, 10 µM BrdU was added for another 16 h. After incubation, the cells were washed with 1x PBS and then collected by centrifugation at 453 x *g* for 5 min. After drying at 55°C, the cell pellets were fixed with FixDenat at 25°C for 30 min, probed with mouse-anti-BrdU-POD for 1.5 h and visualized with TMB substrate for 5 min. The reaction was stopped with the addition of H_2_SO_4_ on a shaker (18 x *g*). The absorbance was detected at a wavelength of 450 nm using an MRX microtiter plate reader.

### Inhibitor Experiments

Cells were pretreated with specific inhibitors for 1 h and then treated with 100 nM ET-3 for the indicated time course. Specific inhibitors, such as BQ610, BQ788, U0126, SB203580, SP600125, AG490, Compound C, Ro318220, SKI-II, and GW4869 (ETAR antagonist, ETBR antagonist, MEK1/2 inhibitor, p38 MAPK inhibitor, JNK inhibitor, JAK2/STAT3 inhibitor, AMPK inhibitor, PKC inhibitor, SphK inhibitor, and SMase2 inhibitor), were used. All chemicals were dissolved in DMSO at a final concentration of 0.1% and dosage following previous studies (28–31).

### Western Blot Analysis

As previously described in detail (27, 30), Western blot analyses were performed with supernatant fractions of preadipocytes. An aliquot of 75 μg of supernatant protein was subjected to 12% SDS-PAGE and prepared with gel loading buffer containing 100 mM Tris-HCl (pH 6.8), 4% SDS, 20% glycerol, 0.2% bromophenol blue, and 10% β-mercaptoethanol. The separated proteins were blotted onto Immobilon-NC membranes and then blocked for 1 h at room temperature with 10 mM PBS containing 0.1% Tween 20 (PBST) and 5% nonfat milk. After washing with PBST, primary antibody at a dilution of 1:1000 (~0.2 μg/ml) was added and then incubated overnight at 4°C. After washing with PBST, the secondary antibody at a dilution of 1:2000 (~0.2 μg/ml) was added. The immunoblots of target proteins were visualized by adding Western Lightning Chemiluminescence Reagent Plus (PerkinElmer Life Sciences, Boston MA, USA) for 3 min. Following exposure to Fuji film for 2~3 min, the blots were quantified using the ImageJ system. The data were calculated by integrated optical density (IOD). After normalization to β-actin, the level of intracellular protein was expressed as a multiple of the control unless otherwise noted. We loaded the known amount (75 ug; in the linear range) of total protein lysates onto the gel according to the standard curve of bovine serum albumin.

### Statistical Analysis

All data were expressed as the mean ± SEM with sample sizes of 3~4. As described in detail previously (27), the statistical analyses were performed using SigmaStat (Jandel Scientific, Palo Alto, CA). Student’s t-test was used for single variable comparisons. One-way ANOVA followed by the Student-Newman-Keuls multiple range test was used to examine differences among multiple groups. Differences were considered significant at p < 0.05. The statistical power was 0.97-1.

## Results

### Effect of ET-3 on Preadipocyte Growth *via* ET_A_R but Not ET_B_R

**Figure 1:**
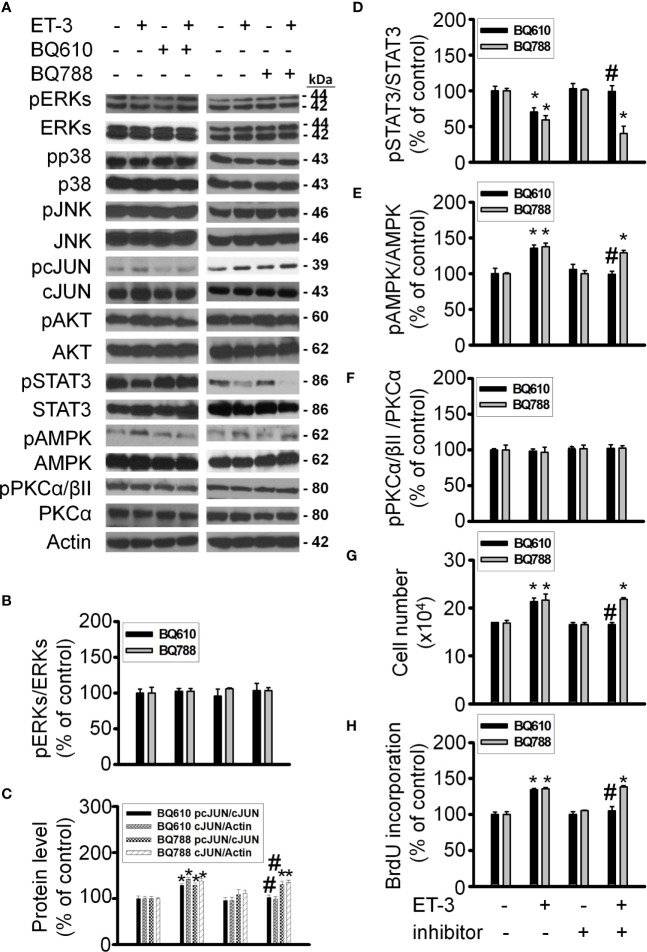
ET_A_R antagonist BQ610 (but not ET_B_R antagonist BQ788) blocked ET-3-altered proliferation and ET-3 receptor activity. Detections of protein expression and cell proliferation in 3T3-L1 preadipocytes treated with inhibitor or ET-3: **(A)** Gel bands in a western blot. **(B–F)** Bar graphs show the analysis of gel bands for normalization to actin; **(G, H)** Cell proliferation measurements by trypan blue staining and BrdU incorporation. All data are expressed as the mean ± SEM of three independent experiments. *p < 0.05 vs. the control; ^#^ *p* < 0.05, ET-3 vs. BQ610 + ET-3.

ET-3 was reported to regulate cell growth in mammalian cancer cells through the ET_B_R-dependent response (32–34). To evaluate the subtype of endothelin receptor that is involved in the effect of ET-3 on preadipocyte growth, we pretreated 3T3-L1 preadipocytes with an ET_A_R or ET_B_R antagonist (BQ610 or BQ788) for 1 h and then treated them with ET-3 (100 nM) (**Figure 1**). We found that the ET_A_R antagonist BQ610, but not the ET_B_R antagonist BQ788, prevented the protein phosphorylation changes stimulated by ET-3, including those of the AMPK, c-JUN, and STAT3 proteins (**Figures 1A–F**). However, ET-3 alone increased the total c-JUN protein levels and pretreatment with BQ610 blocked the ET-3-induced increases in total c-JUN protein levels. We assessed the number of cells (**Figure 1G**) and proliferation of cells (**Figure 1H**) and found that pretreatment with BQ610 prevented ET-3-induced increases in both cell number and BrdU incorporation. These data suggest that ET-3-altered preadipocyte growth is involved in the ET_A_R pathway.

### Effect of ET-3 on Preadipocyte Growth Through ERK and JNK

**Figure 2:**
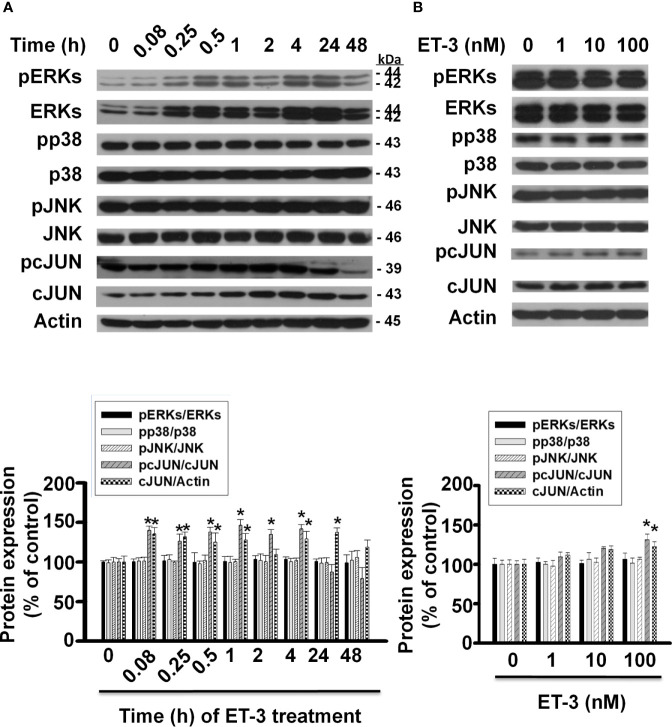
Effects of ET-3 on MAPK protein levels in 3T3-L1 preadipocytes. Detection of protein expression in 3T3-L1 preadipocytes treated with ET-3: **(A)** at different time points measured by western blots and the results were assessed at time 0; and **(B)** at different dosages measured by western blots and the results were normalized to actin. All data are expressed as the mean ± SEM of three independent experiments. *p < 0.05, vs. the controls.

**Figure 3:**
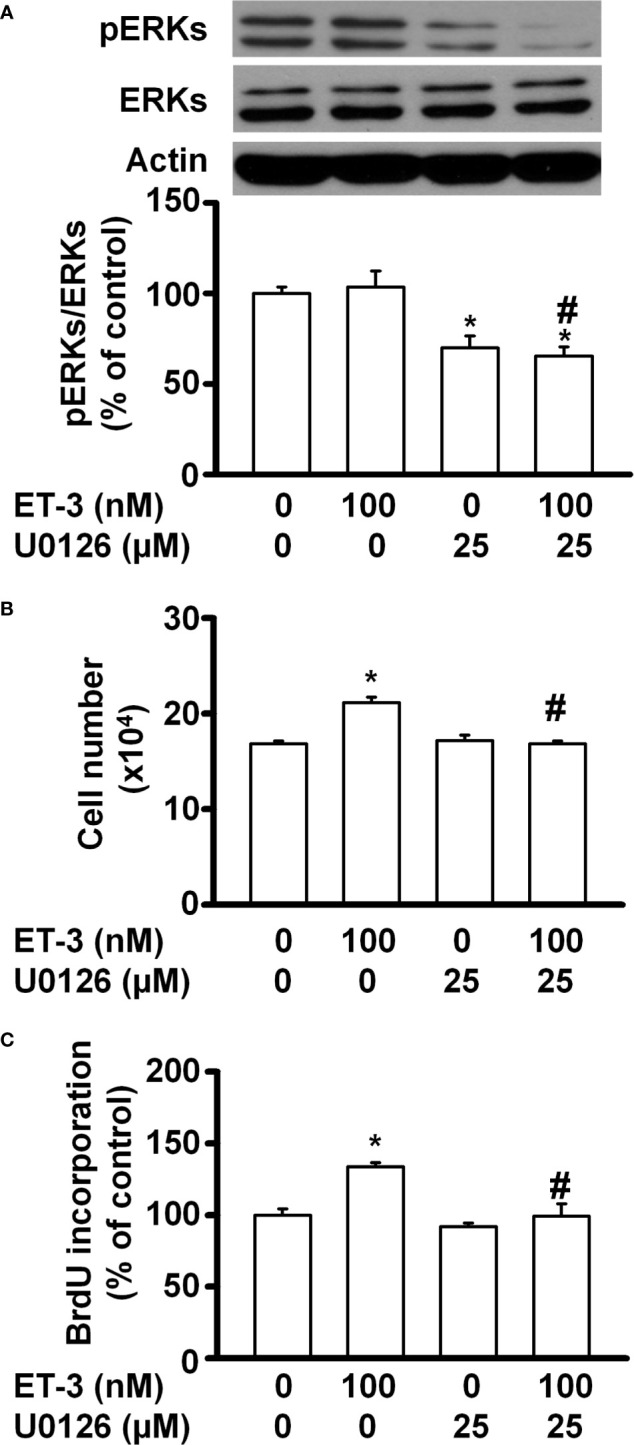
Specific inhibitors of ERK MAPK, such as U0126, affected ET-3-induced cell growth in 3T3-L1 preadipocytes. Detection of protein expression and cell proliferation in 3T3-L1 preadipocytes treated with U0126 or ET-3: **(A)** Data were analysed by western blots, and the results were normalized to actin. **(B, C)** Cell proliferation measurements by trypan blue staining and BrdU incorporation. All data are expressed as the mean ± SEM of three independent experiments. *p < 0.05 vs. the controls; ^#^ *p* < 0.05, ET-3 vs. U0126 + ET-3.

The MAPK pathways involve multiple protein kinases and play an important role in the regulation of cell proliferation in many mammalian cell types (35–37). MAPK families have three members: extracellular signal-regulated kinase (ERK), Jun kinase (JNK) and p38 MAPK. In adipocytes, ET-1 has been reported to regulate cell proliferation, adipogenesis, and other cell responses though the MAPK pathway (13, 38). ET-3 is an endothelin family member that regulates colon carcinoma cell proliferation and melanocyte growth. We found that ET-3 stimulated preadipocyte proliferation by changing the cell number and BrdU incorporation (**Figure 1**). To evaluate whether ET-3 alters preadipocyte growth *via* the MAPK pathway, we pretreated preadipocytes with MAPK protein inhibitors (U0126, SP600125, and SB203580) and then treated preadipocytes with or without ET-3 (100 nM) (**Figures 2**, **3** and **Supplementary Figures 1, 2**). According to the time-dependent variations in protein levels (**Figure 2**), ET-3 altered JNK/c-JUN protein expression. However, ET-3 treatment did not change the pERK/ERK protein percentage, which indicates that ET-3 alters the total ERK protein amount (as indicated in the time course). Furthermore, we determined whether ET-3 affects the MAPK pathway, and the data showed that U0126 (MEK1/ERK inhibitor) and SP600125 (JNK/c-JUN inhibitor) blocked ET-3-stimulated cell numbers and BrdU incorporation (**Figure 3** and **Supplementary Figure 1**). However, ET-3 did not change p38 MAPK protein expression (**Supplementary Figure 2A**). These data suggest that MEK1/ERK and JNK/c-JUN are needed for ET-3-altered preadipocyte growth.

### Effect of ET-3 on Preadipocyte Growth *via* the JAK2/STAT3 Pathway

**Figure 4:**
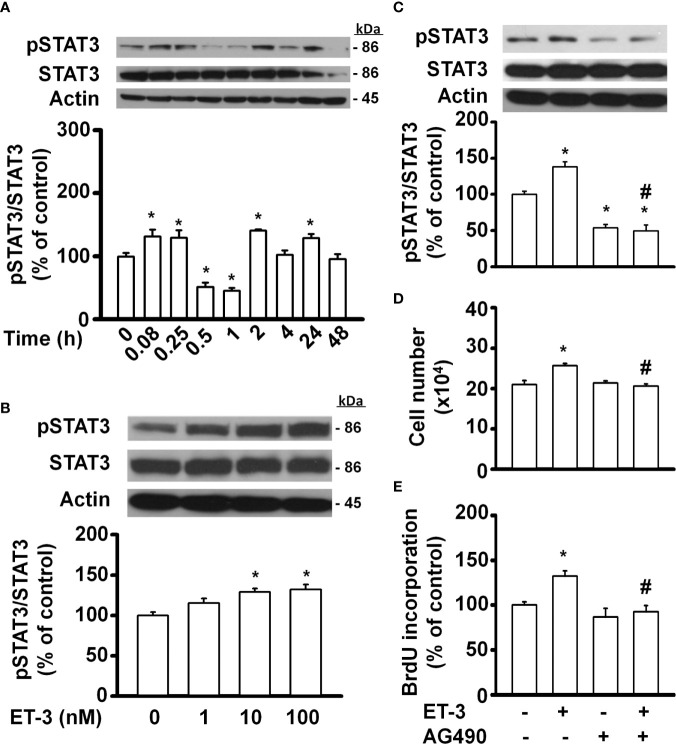
Specific inhibitors of STAT3, such as AG490, antagonized ET-3-induced cell growth in 3T3-L1 preadipocytes. Detection of protein expression and cell proliferation in 3T3-L1 preadipocytes treated with AG490 or ET-3: **(A)** at different time points measured by western blots and the results were assessed at time 0; **(B)** at different dosages measured by western blots and the results were normalized to actin; **(C)** data were analysed by western blots and the results were normalized to actin and **(D, E)** cell proliferation measurements by trypan blue staining and BrdU incorporation. All data are expressed as the mean ± SEM of three independent experiments. *p < 0.05 vs. the controls; ^#^ *p* < 0.05, ET-3 vs. AG490 + ET-3.

Signal transducer and activator of transcription (STAT)-3 is an important transcription factor involved in many biological processes, including proliferation, development, differentiation, inflammation, and apoptosis (35, 39–41). In fat cells, STAT3 regulates adipocyte differentiation and adipogenesis (28, 41). Whether ET-3-altered preadipocyte growth is related to the STAT3 pathway was assessed (**Figure 4**). We found that ET-3 increased the phosphorylation of STAT3 in time- and dose-dependent manners (**Figures 4A, B**). Additionally, we observed that ET-3 treatment increased the phosphorylation of STAT3 (**Figure 4C**). Pretreatment with AG490 reduced the basal phosphorylation of STAT3 and suppressed ET-3-altered pSTAT3 protein expression. However, AG490 had no effects on the total STAT3 protein level, cell number, or BrdU incorporation (**Figures 4C–E**). Compared to ET-3 treatment, AG490 significantly reduced the ET-3-altered increases in both cell number and BrdU incorporation.

### Effect of ET-3 on Preadipocyte Growth *via* the Sphingosine Kinase (SphK) and Sphingomyelinase (SMase) 2 Pathways

**Figure 5:**
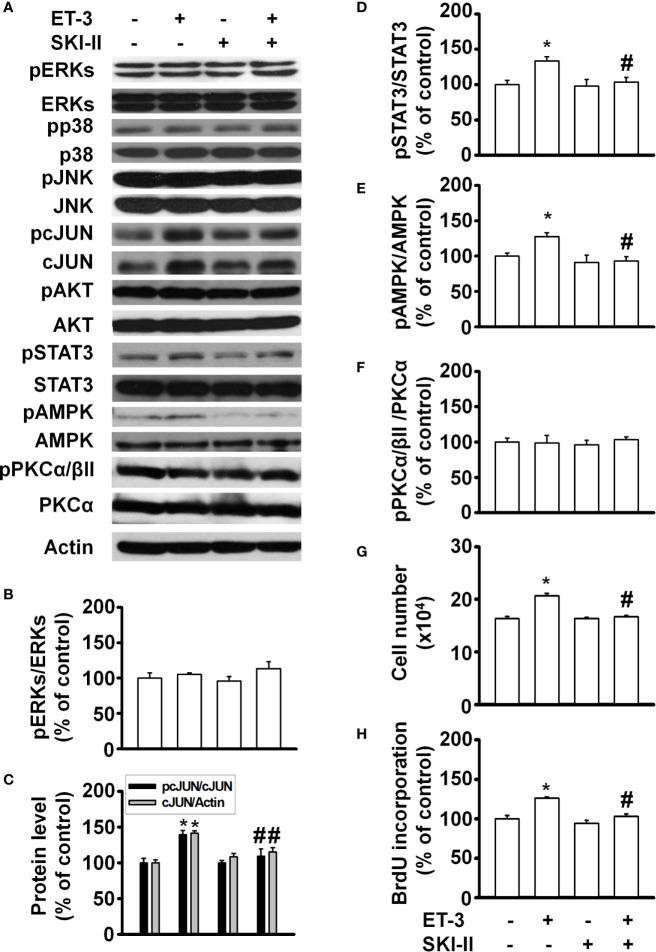
Sphingosine kinase (SphK) inhibitor SKI-II modified ET-3-induced cell growth in 3T3-L1 preadipocytes. Detection of protein expression and cell proliferation in 3T3-L1 preadipocytes treated with SKI-II or ET-3: **(A)** gel bands in a western blot assay. **(B–F)** bar graphs showing the analysis of gel bands for normalization to actin; and **(G, H)** cell proliferation measurements by trypan blue staining and BrdU incorporation. All data are expressed as the mean ± SEM of three independent experiments. *p < 0.05 vs. the controls; ^#^ *p* < 0.05, ET-3 vs. SKI-II + ET-3.

SphK kinase induces many cellular responses, including proliferation, inhibition of apoptosis, formation of actin stress fibres, and stimulation of adherent junctions (29, 42–45). To determine whether ET-3 alters preadipocyte growth *via* the SphK pathway, we pretreated cells with the SphK inhibitor SKI-II and then treated them with 100 nM ET-3 (**Figure 5**). The results showed that SKI-II blocked ET-3-altered cell number (**Figure 5G**), BrdU incorporation (**Figure 5H**), and phosphorylations of c-JUN (**Figure 5C**), STAT3 (**Figure 5D**), and AMPK (**Figure 5E**) proteins.

Recently, the SMase pathway was reported to mediate individual or cooperative effects on ceramide synthesis, thus leading to ceramide signalling and cascade reactions for downstream cell responses, including the regulation of cell growth, viability, and differentiation (46). To determine whether ET-3 alters preadipocyte growth *via* the SMase pathway, we pretreated cells with the SMase inhibitor GW4869 for 1 h and then treated them with or without ET-3 (100 nM) for 1 h (**Supplementary Figure 3**). The data showed that GW4869 blocked the ET-3-altered cell number, BrdU incorporation, and phosphorylations of c-JUN, STAT3, and AMPK proteins. However, treatment with either SKI-II or GW4869 alone had no effect on the number of cells or BrdU incorporation. These data indicated that ET-3-altered preadipocyte growth involves the SphK and SMase pathways.

### Cell Type Dependence of ET-3

**Figure 6:**
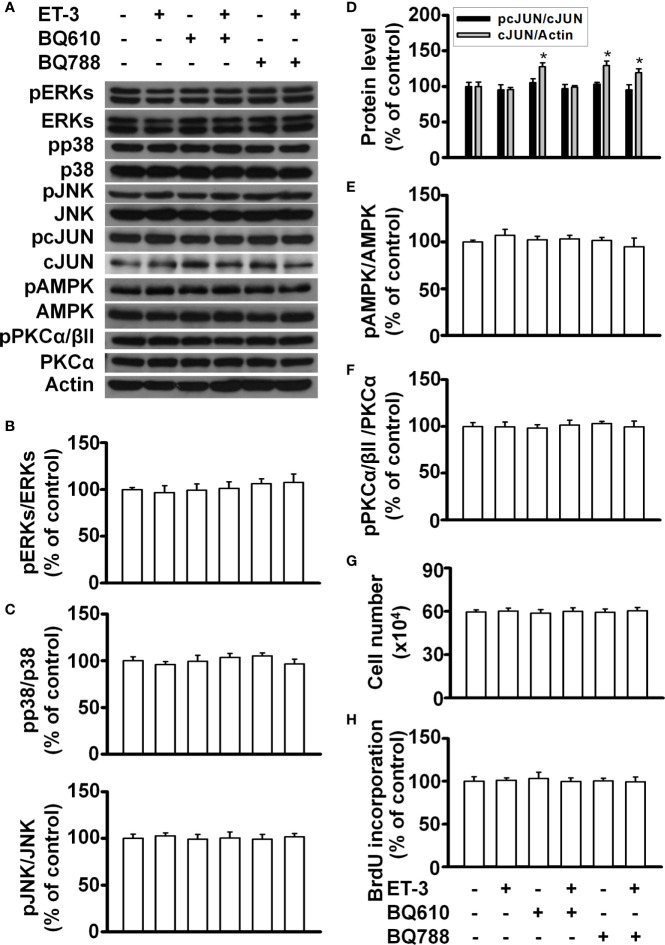
Effects of ET-3 signalling cascades in HIB1B brown preadipocytes. Detection of protein expression and cell proliferation in HIB1B preadipocytes treated with inhibitor or ET-3: **(A)** gel bands in a western blot; **(B–F)** bar graphs show the analysis of gel bands for normalization to actin; and **(G, H)** cell proliferation measurements by trypan blue staining and BrdU incorporation. All data are expressed as the mean ± SEM of three independent experiments. *p < 0.05, vs. the controls.

**Figure 7:**
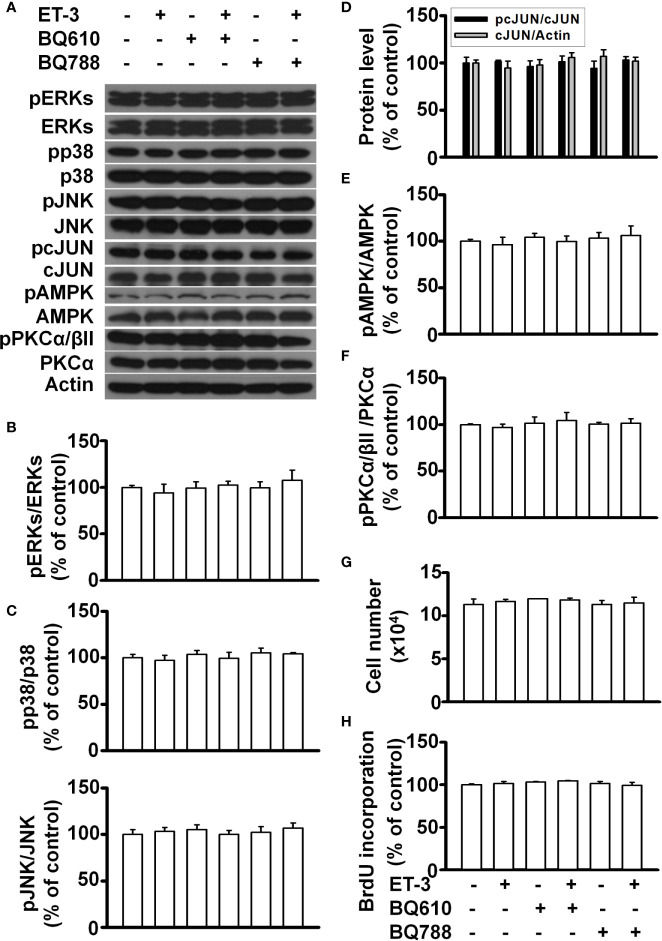
Effects of ET-3 signalling cascades in D12 beige preadipocytes. Detection of protein expression and cell proliferation in HIB1B preadipocytes treated with inhibitor or ET-3: **(A)** gel bands in a western blot; **(B–F)** bar graphs show the analysis of gel bands for normalization to actin; and **(G, H)** cell proliferation measurements by trypan blue staining and BrdU incorporation. All data are expressed as the mean ± SEM of three independent experiments.

To determine whether the ET-3-induced stimulation of preadipocyte growth is dependent on the type of preadipocytes, we treated murine HIB1B brown preadipocytes or D12 beige preadipocytes with different doses of ET-3 (**Figures 6**, **7**). None of the ET-3, BQ610, BQ788, or their combinations altered the protein levels of pERK, pp38, pJNK, pAMPK, or pPKC in HIB1B or D12 cells relative to the controls or ET-3-treated cells (**Figures 6A–F** and **7A–F**). In addition, ET-3 alone significantly decreased the pcJUN protein levels, which was reversed by BQ610 in HIB1B preadipocytes (**Figure 6D**). Although ET-3 slightly increased the number of HIB1B brown preadipocytes (**Figure 6G**) and D12 beige preadipocytes (**Figure 7G**) after 48 h of treatment, no statistical significance was observed. ET-3 did not significantly alter the magnitude of BrdU incorporation in HIB1B (**Figure 6H**) and D12 cells (**Figure 7H**). These results indicated a preadipocyte type-dependent effect of ET-3 on cell growth.

## Discussion

Our experimental data showed that ET-3 induced increases in the cell number and BrdU incorporation in 3T3-L1 preadipocytes. We also found that PKC was not involved in ET-3-stimulated preadipocyte growth, which may help explain the different effects of ET-1. Interestingly, we observed that pretreatment with BQ610 (but not BQ788) prevented ET-3-induced increases in cell number and BrdU incorporation in preadipocytes. This finding is different from previous studies showing that ET-3 regulates cell growth of melanocytes and intestinal cells though the ETBR pathway. Therefore, we hope to use the results of this study to prove that ET-3 acts on the growth of various cell types with receptor type dependency.

In most reports, ETs affect many physiological and pathological responses, including vasoconstriction, ROS production, platelet activation, coagulation, lipid metabolism, inflammation, cell growth, and apoptosis (3, 12, 13, 25). In fat cells, ET-1 was demonstrated to modulate glucose transporter (GLUT) 1 transcription, adiponectin and resistin secretion, lipolysis, and proliferation though the ET_A_R pathway (30, 31). However, few studies have examined the role of ET-2 or ET-3 in fat cells. Current research on ET-3 is mainly focused on the development of neural crest-derived epidermal melanin (15). Moreover, some studies have recently shown that this molecule is related to colon cancer cell proliferation. We attempted to identify the downstream signalling transducers of ET-3 in fat cells that are involved in stimulating preadipocyte growth. AMP-activated protein kinase (AMPK) has been reported in a variety of physiological and pathological conditions, where it plays a critical role, including regulating growth and lipid metabolism (47). AMPK also regulates cellular processes related to autophagy and cell polarity (48). AMPK has been reported in a variety of physiological and pathological conditions where it plays a critical role in regulating growth and lipid metabolism (47). According to the time-dependent AMPK protein levels (**Supplementary Figure 4**), ET-3 specifically increased AMPK phosphorylation in a time-dependent manner, and treatment with an AMPK inhibitor (Compound C) blocked ET-3-induced phosphorylation of AMPK and preadipocyte growth (**Supplementary Figure 4**). These data showed that AMPK may play an important role in preadipocyte growth. PKC mediates many biological processes, including cell cycle regulation and cell survival (46). ET-1 stimulated preadipocyte proliferation (27) and adipocyte differentiation in 3T3-L1 preadipocytes. According to the time-dependent changes in PKC protein levels, ET-3 did not affect PKC protein phosphorylation. In parallel, treatment with a PKC inhibitor (Ro318220) did not block ET-3-induced preadipocyte growth (**Supplementary Figure 5**). These data indicated that ET-3 did not alter preadipocyte growth *via* the PKC pathway. We observed that specific inhibitors of ERK (U0126), JNK (SP600125), JAK2 (AG490), AMPK (Compound C), SphK (SKI-II), and SMase2 (GW4869) significantly prevented ET-3-induced increases in both cell number and BrdU incorporation. Some of the inhibitors also antagonized the ET-3-induced increases in the pcJUN, pSTAT3, or pAMPK protein levels. However, ET-3 did not stimulate the phosphorylation of ERK or p38 MAPK. Neither p38 MAPK inhibitor (SB203580) nor PKC inhibitor (Ro318220) altered the ET-3 effect on preadipocyte growth. These observations suggest that the stimulatory effect of ET-3 on the growth of 3T3-L1 preadipocytes is mediated by a pathway that requires the activation of JNK/c-JUN, STAT3 AMPK, SphK, and SMase2 and is independent of PKC and p38 MAPK pathways.

**Figure 8:**
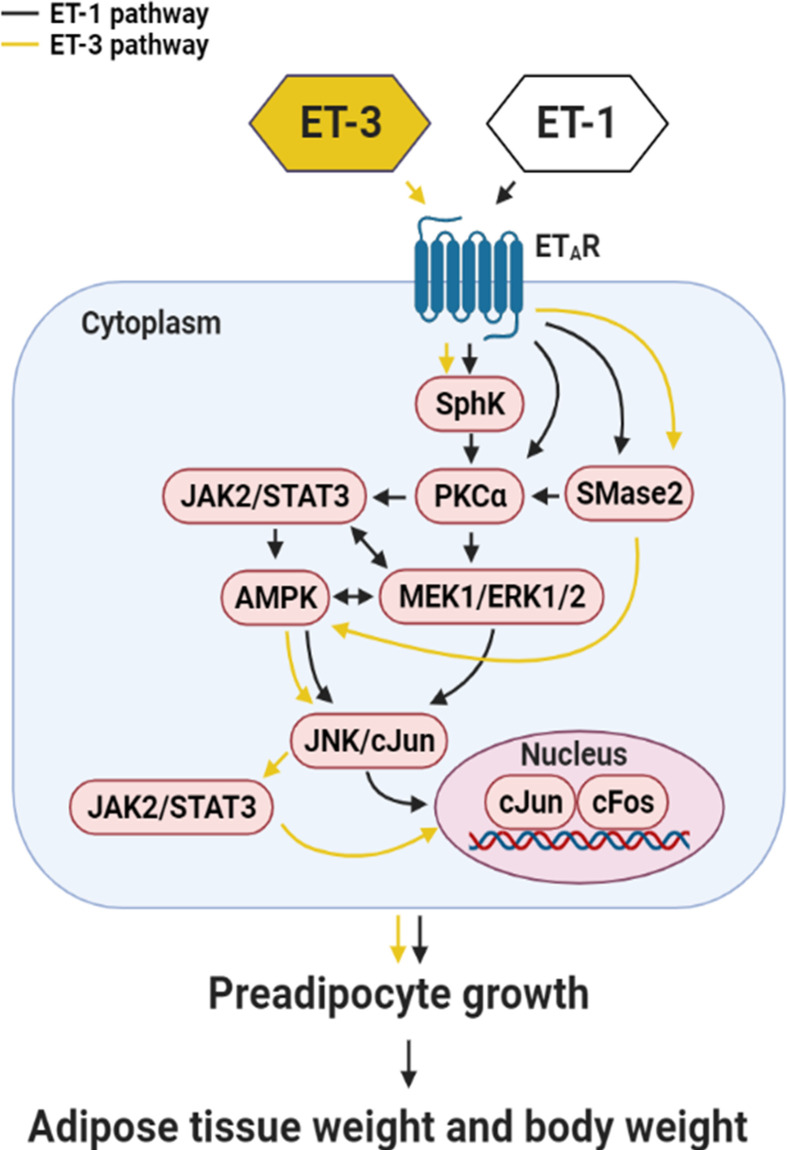
Summary of the mechanism by which ET-3 differs from ET-1 to regulate the growth of 3T3-L1 preadipocytes. ET-3 signalling to induce preadipocyte growth is mediated through the AMPK, JNK/c-JUN, JAK2/STAT3, SphK, SMase2, and ET_A_R pathways. Compared with the growth signal of ET-1 in preadipocytes, the signal transduction of ET-3 is independent of the PKC pathway.

Many studies have proven that ERK is directly related to proliferation (36, 37). The data presented here showed that the pERK/ERK percentage did not change significantly, which was possibly because the level of total ERK increased. Therefore, an increase in total ERK will lead to an increase in ERK phosphorylation. In this case, ERK may play a role in maintaining preadipocyte survival. ET-3-altered STAT3 protein phosphorylation has a time-dependent effect, in which STAT3 phosphorylation changes every 2 h. Previous studies have indicated that SOCS3 negatively regulates STAT3 gene expression (49). We speculate that fat cells may have a similar effect, which could be explored in the future. Pretreatment with an AMPK inhibitor (Compound C) blocked the ET-3-stimulated phosphorylation of c-JUN. AMPK may act as the upstream signal transducer of the c-JUN protein. The SphK-mediated pathway and SMase2-mediated pathway prevent the apoptosis of uterine leiomyoma cells and the proliferation of hepatic stellate cells, respectively (29, 50), whereas the SMase2/ceramide-mediated pathway regulates glucose uptake in 3T3-L1 preadipocytes and adipocytes in the absence of ET-1 (51–53). Our data showed that a crosstalk might also occur between SphK/SMase2 and other kinase cascades because pretreatment with the SphK inhibitor SKI-II antagonized the ET-3-induced increases in STAT3, AMPK, and c-JUN phosphorylation and cell growth and because pretreatment with the SMase2 inhibitor GW4869 antagonized the ET-3-induced increases in AMPK and c-JUN phosphorylation and cell growth. The results of this study, together with our previous ET-1 signalling pathways in the regulation of preadipocyte growth, could be used for comparing the differences of growth signals of ET-3 with ET-1 in the regulation of fat cell proliferation (**Figure 8**).

The development of obesity is characterized by adipocyte hyperplasia and hypertrophy and can be regulated by ETs (5). For example, chronic ET-1 infusion caused adipocyte hyperplasia in rats (38). The *in vivo* study was supported by our *in vitro* findings that ET-1 stimulated the growth of murine primary and secondary preadipocytes *via* the ET_A_R pathway (27). In the present study, ET-3 was demonstrated to stimulate 3T3-L1 white preadipocyte growth *via* the ET_A_R pathway. Thus, whether the ET-3-ETAR pathway is like the ET-1-ET_A_R pathway to contribute in the stimulation of adipocyte hyperplasia and thereby maintaining adipostat for body weight control warrants further investigation.

In this study, the effect of ET-3 on the growth of preadipocytes was preadipocyte type-dependent. In particular, ET-3 stimulated the growth of 3T3-L1 white preadipocytes but not HIB1B brown preadipocytes or D12 beige preadipocytes. A semiquantitative real-time PCR assay of the levels of ET_A_R and ET_B_R mRNAs in all three types of preadipocytes showed that the 3T3-L1 cell line expressed greater ET_A_R mRNA levels than the HIB1B and D12 cell lines (**Supplementary Figure 6**). Interestingly, all types of cells expressed nondetectable ET_B_R mRNA levels. Whether the different levels of ET_A_R mRNA expression explain the more marked effect of ET-3 on 3T3-L1 cell growth and lack of significant effect of ET-3 on HIB1B and D12 cell growth requires more thorough demonstrations in the further study.

The limitations of the present study are as follows. First, all experiments were performed using only inhibitors. For example, in addition to being a selective inhibitor of JAK2/STAT3, AG490 can act as a selective inhibitor of the EGF receptor (EGFR) tyrosine kinase, JAK3/AP-1, and JAK3/MAPK pathways. Accordingly, a well-designed JAK2/STAT3 gene silencing experiment may help strengthen the specific effect of AG490 used in the study. Alternatively, further study to demonstrate whether any of EGFR and JAK3/AP-1 proteins are necessary for ET-3-mediated growth of preadipocytes may help strengthen the specific effect of AG490. Second, when we set up the experiment to examine the simultaneous administration of ET-1, ET-2, and ET-3 to 3T3-L1 cells, we found that neither ET-2 nor ET-3 enhanced the ET-1-induced increase in the cell number of 3T3-L1 preadipocytes after 48 h of treatment (**Supplementary Figure 7**). Because the potency of ET-1 to induce an increase in the cell number was greater than that of ET-3, the crosstalk of signal cascades between ET-1 and ET-3 was difficult and not demonstrated in this study. Interestingly, ET-2 had no significant effect to stimulate the growths of 3T3-L1, HIB1B, and D12 preadipocytes. Third, because the effects of ET-1 on the growth and signal cascades of 3T3-L1 preadipocytes were demonstrated (27), we did not set up the ET-1 treatment as the positive group in all experiments of the study. Fourth, because ET-3 was not observed to stimulate the growth of D12 and HIB1B cells (**Supplementary Figure 8**), similar experiments as those shown in **Figures 1**–**5** were not performed in this study. Fifth, when primary cells (stromal cell fraction) derived from mouse epididymal adipose tissues were treated with 100 nM ET-3, we observed that ET-3 tended to induce increases in the cell number (p = 0.08) and cell viability (p = 0.07) (**Supplementary Figure 9**). Such a tendency was reversed after BQ610 but not BQ788 treatment.

## Conclusions

We concluded that the effect of ET-3 on preadipocyte growth is dependent on the JAK2/STAT3, c-JUN, AMPK, SphK, SMase2, and ET_A_R pathways and independent of the p38 MAPK, PKC, ceramide synthase (CerS), and ET_B_R pathways (**Figure 8**). A certain crosstalk occurs among the ET_A_R downstream signalling cascades. The ET-3-induced stimulation of fat cell growth varies with the type of preadipocytes. ET-3 exhibits somewhat different signals from ET-1 (27) to stimulate preadipocyte proliferation through modulations of ERK- and PKC-independent pathways. The results of this study may help explain how different ET isoforms mediate fat cell activity and fat cell-associated disease and how ETs possess multiple effects in fat cells.

## Data Availability Statement

The original contributions presented in the study are included in the article/**Supplementary Material**. Further inquiries can be directed to the corresponding authors.

## Author Contributions

Conceptualization, L-JS, Y-YL, C-PC, Y-CK, Y-WT, and Y-HK. Data collection, A-CS and L-JS. Formal analysis, A-CS and L-JS. Methodology, A-CS and L-JS. Validation, A-CS, L-JS, Y-YL, C-PC, Y-HK, and P-JH. Writing—original draft, A-CS, Y-HK, and P-JH. Writing—review and editing, Y-HK and P-JH. All authors contributed to the article and approved the submitted version.

## Funding

This study was supported by grants from the Ministry of Science and Technology, Taiwan (MOST-106-2320-B-008-008-MY3 and MOST-109-2320-B-008-001-MY3); National Central University and Landseed Hospital Joint Research (NCU-LSH-107-B-006, -108-B-009; -109-B-00); and Research Fund of the Taoyuan Armed Forces General Hospital (AFTYGH-10612, -10716, -10717, -10813, and -10814; AFTYGH-D-109-018 and AFTYGH-D-110-032). This study was supported by grants from the Ministry of Science and Technology, Taiwan to Y-HK; National Central University and Landseed Hospital Joint Research to Y-HK; and Research Fund of the Taoyuan Armed Forces General Hospital to Y-HK, Y-YL, Y-WT, L-JS and P-JH.

## Conflict of Interest

The authors declare that the research was conducted in the absence of any commercial or financial relationships that could be construed as a potential conflict of interest.
