# Supplementary material for: Investigation of the Molecular Mechanisms by Which Endothelin-3 Stimulates Preadipocyte Growth
Source: Front Endocrinol (Lausanne). 2021 May 21;12:661828. doi: 10.3389/fendo.2021.661828 (PMC8176213; doi:10.3389/fendo.2021.661828)
Supplement: Supplementary file 1 [file DataSheet_1.docx]

Supplementary Material

# Supplementary Materials

**Supplementary Figure 1.** Specific inhibitors of JNK MAPK, such as SP600125, antagonized ET-3-induced cell growth in 3T3-L1 preadipocytes. Detection of protein expression and cell proliferation in the 3T3-L1 preadipocytes treated with SP600125 or ET-3: (**A**) Data were analysed by western blots and normalized to actin. Scale bar = 1 cm; (**B-C**) Cell proliferation measurements by trypan blue staining and BrdU incorporation. All data are expressed as the mean ± SEM of three independent experiments. * *p* <0.05 vs. the controls; # *p* < 0.05, ET-3 vs. SP600125 + ET-3

**Supplementary** **Figure 2.** Specific inhibitors of p38 MAPK, such as SB203580, had no effect on ET-3-induced cell growth in 3T3-L1 preadipocytes.Detection of protein expression and cell proliferation in the 3T3-L1 preadipocytes treated with SB203580 or ET-3: (**A**) Data were analysed by western blots and normalized to actin. Scale bar = 1 cm; (**B-C**) Cell proliferation measurements by trypan blue staining and BrdU incorporation. All data are expressed as the mean ± SEM of three independent experiments. * *p* <0.05 vs. the controls; # *p* < 0.05, ET-3 vs. SB203580 + ET-3

**Supplementary** **Figure 3.** The sphingomyelinase (SMase) inhibitor GW4869 modified ET-3-induced cell growth in 3T3-L1 preadipocytes. Detection of protein expression and cell proliferation in the 3T3-L1 preadipocytes treated with GW4869 or ET-3: (**A**) Gel bands in a western blot. Scale bar = 1 cm; (**B-F**) Bar graphs show the analysis of gel bands for normalization to actin; (**G-H**) Cell proliferation measurements by trypan blue staining and BrdU incorporation. All data are expressed as the mean ± SEMof three independent experiments. * *p* <0.05 vs. the controls; # *p* < 0.05, ET-3 vs. GW4869 + ET-3

**Supplementary** **Figure 4.** Specific inhibitors of AMPK, such as Compound C, antagonized ET-3-induced cell growth in 3T3-L1 preadipocytes. Detection of protein expression and cell proliferation in the 3T3-L1 preadipocytes treated with compound C or ET-3: (**A**) At different time points measured by western blots and the results assessed at time 0; (**B**) At different dosages measured by western blots and the result normalized to actin; (**C**) Data were analysed by western blots and the result normalized to actin. Scale bar = 1 cm; (**D-E**) Cell proliferation measurements by trypan blue staining and BrdU incorporation. All data are expressed as the mean ± SEM deviation of three independent experiments. * *p* <0.05 vs. the controls; # *p* < 0.05, ET-3 vs. Compound C + ET-3

**Supplementary Figure 5.** ET-3 had no significant effect on PKC protein in 3T3-L1 preadipocytes.Detection of protein expression and cell proliferation in the 3T3-L1 preadipocytes treated with Ro318220 or ET-3: (**A**) At different time points measured by western blots and the results assessed at time 0; (**B**) At different dosages measured by western blots and the results normalized to actin; (**C**) Data were analysed by western blots and the result normalized to actin. Scale bar = 1 cm; (**D-E**) Cell proliferation measurements by trypan blue staining and BrdU incorporation. All data are expressed as the mean ± SEM of three independent experiments. * *p* <0.05 vs. the controls; # *p* < 0.05, ET-3 vs. Ro318220 + ET-3

**Supplementary Figure 6.** Expression of ETAR and ETBR genes in 3T3-L1, HIB1B, and D12 cells after analysis of RT-PCR (A) and real-time PCR (B). Data were expressed as means ± SEM. *, p < 0.05, vs. 3T3-L1

**Supplementary Figure 7.**  Differential effects of endothelin (ET)-1, ET-2, and ET-3 on the cell number (A), BrdU incorporation (B), and cell viability (C) of 3T3-L1 preadipocytes were observed. The cell number, BrdU incorporation, and cell viability were respectively detected after 48 h, 18 h, and 48 h of 100-nM ET treatments. *, P < 0.05, vs the control

**Supplementary Figure 8.**  Differential effects of endothelin (ET)-1, ET-2, and ET-3 on the cell number and cell viability of 3T3-L1 white preadipocytes, HIB1B brown preadipocytes, and D12 beige preadipocytes were observed after 48 h of 100-nM ET treatment. *, P < 0.05, vs the control

**Supplementary Figure 9.**  Endothelin (ET)-3 at 100 nM tended to stimulate the growth of primary preadipocytes derived from the stromal fraction of mouse epididymal adipose tissues, as indicated by increased cell number (**A**; P = 0.08) and increased cell viability (**B**; P = 0.07). The effect was blocked after 1 μM of BQ610 treatment.

# Supplementary Figures


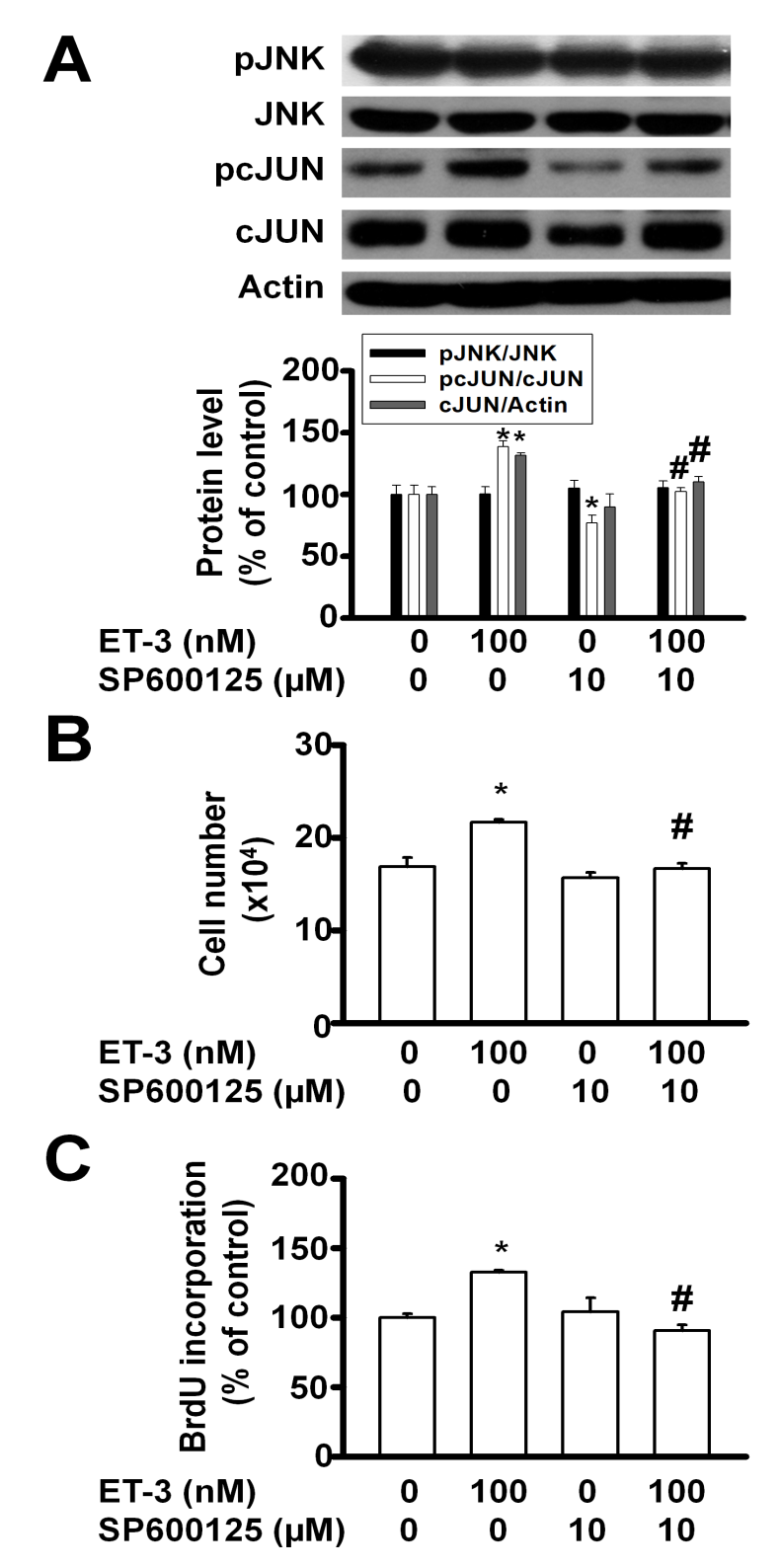


**Supplementary Figure 1.** Specific inhibitors of JNK MAPK, such as SP600125, antagonized ET-3-induced cell growth in 3T3-L1 preadipocytes. Detection of protein expression and cell proliferation in the 3T3-L1 preadipocytes treated with SP600125 or ET-3: (**A**) Data were analysed by western blots and normalized to actin. Scale bar = 1 cm; (**B-C**) Cell proliferation measurements by trypan blue staining and BrdU incorporation. All data are expressed as the mean ± SEM of three independent experiments. * *p* <0.05 vs. the controls; # *p* < 0.05, ET-3 vs. SP600125 + ET-3


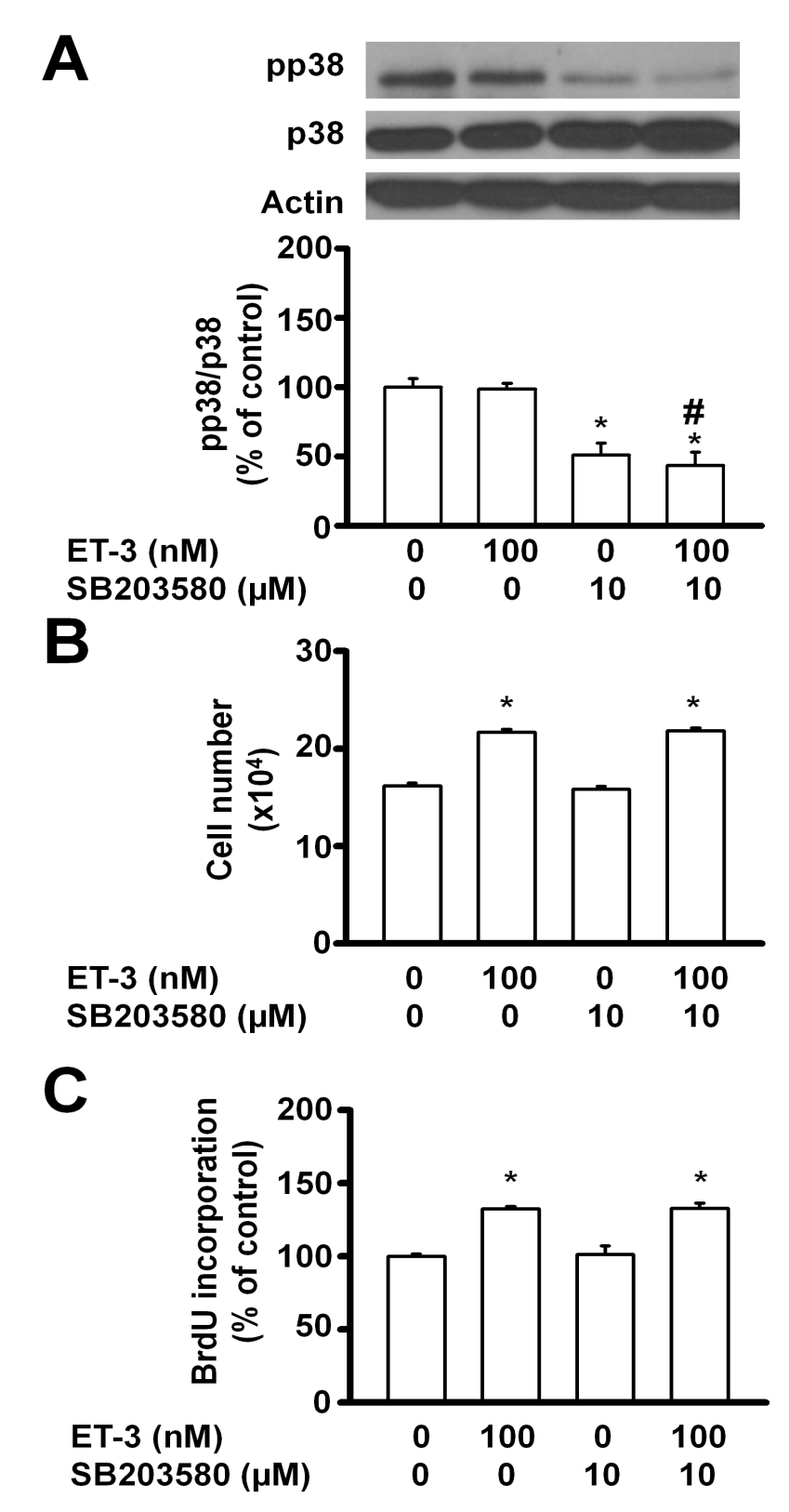


**Supplementary Figure 2.** Specific inhibitors of p38 MAPK, such as SB203580, had no effect on ET-3-induced cell growth in 3T3-L1 preadipocytes. Detection of protein expression and cell proliferation in the 3T3-L1 preadipocytes treated with SB203580 or ET-3: (**A**) Data were analysed by western blots and normalized to actin. Scale bar = 1 cm; (**B-C**) Cell proliferation measurements by trypan blue staining and BrdU incorporation. All data are expressed as the mean ± SEM of three independent experiments. * *p* <0.05 vs. the controls; # *p* < 0.05, ET-3 vs. SB203580 + ET-3


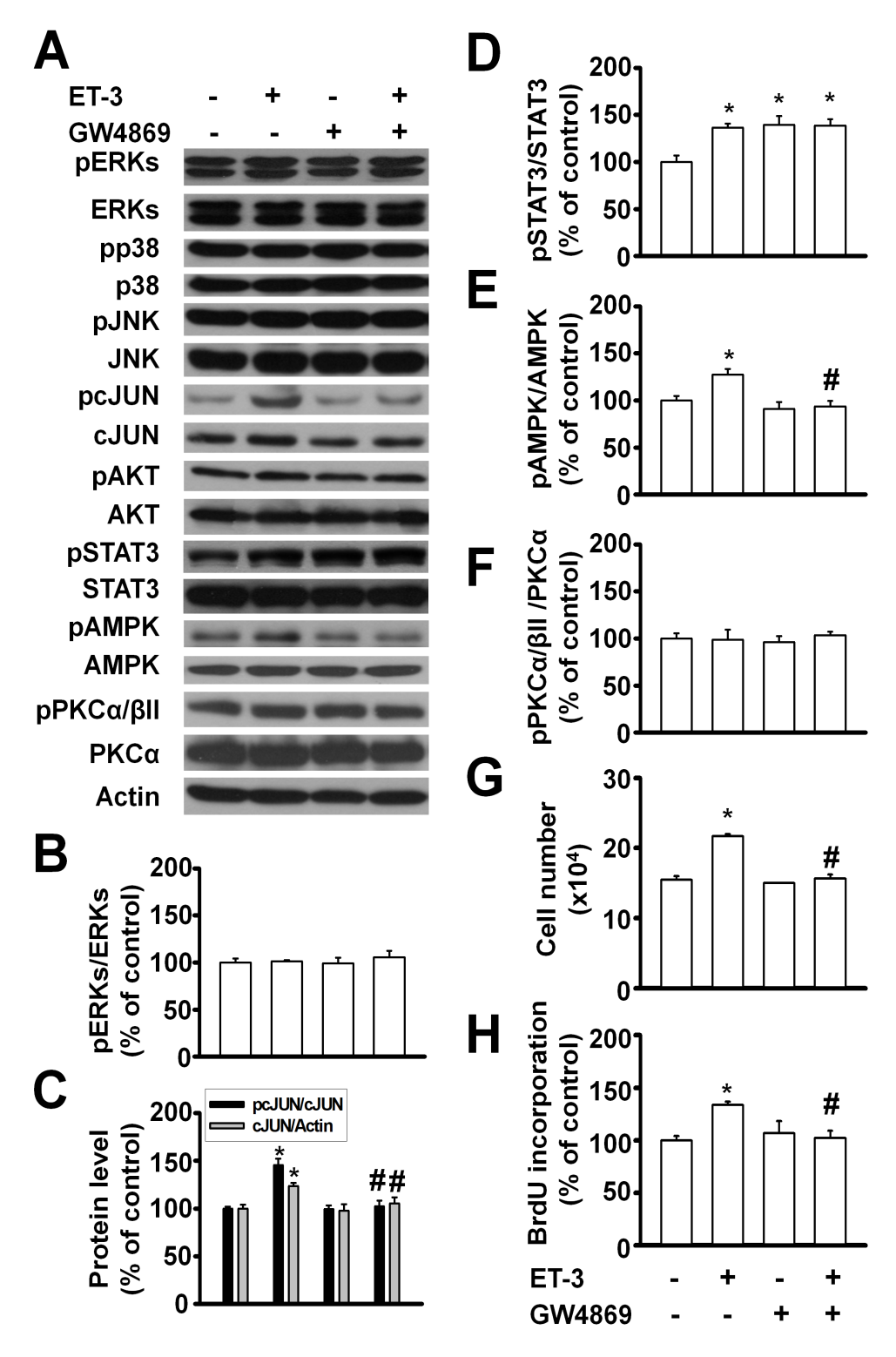


**Supplementary Figure 3.** The sphingomyelinase (SMase) inhibitor GW4869 modified ET-3-induced cell growth in 3T3-L1 preadipocytes. Detection of protein expression and cell proliferation in the 3T3-L1 preadipocytes treated with GW4869 or ET-3: (**A**) Gel bands in a western blot. Scale bar = 1 cm; (**B-F**) Bar graphs show the analysis of gel bands for normalization to actin; (**G-H**) Cell proliferation measurements by trypan blue staining and BrdU incorporation. All data are expressed as the mean ± SEMof three independent experiments. * *p* <0.05 vs. the controls; # *p* < 0.05, ET-3 vs. GW4869 + ET-3


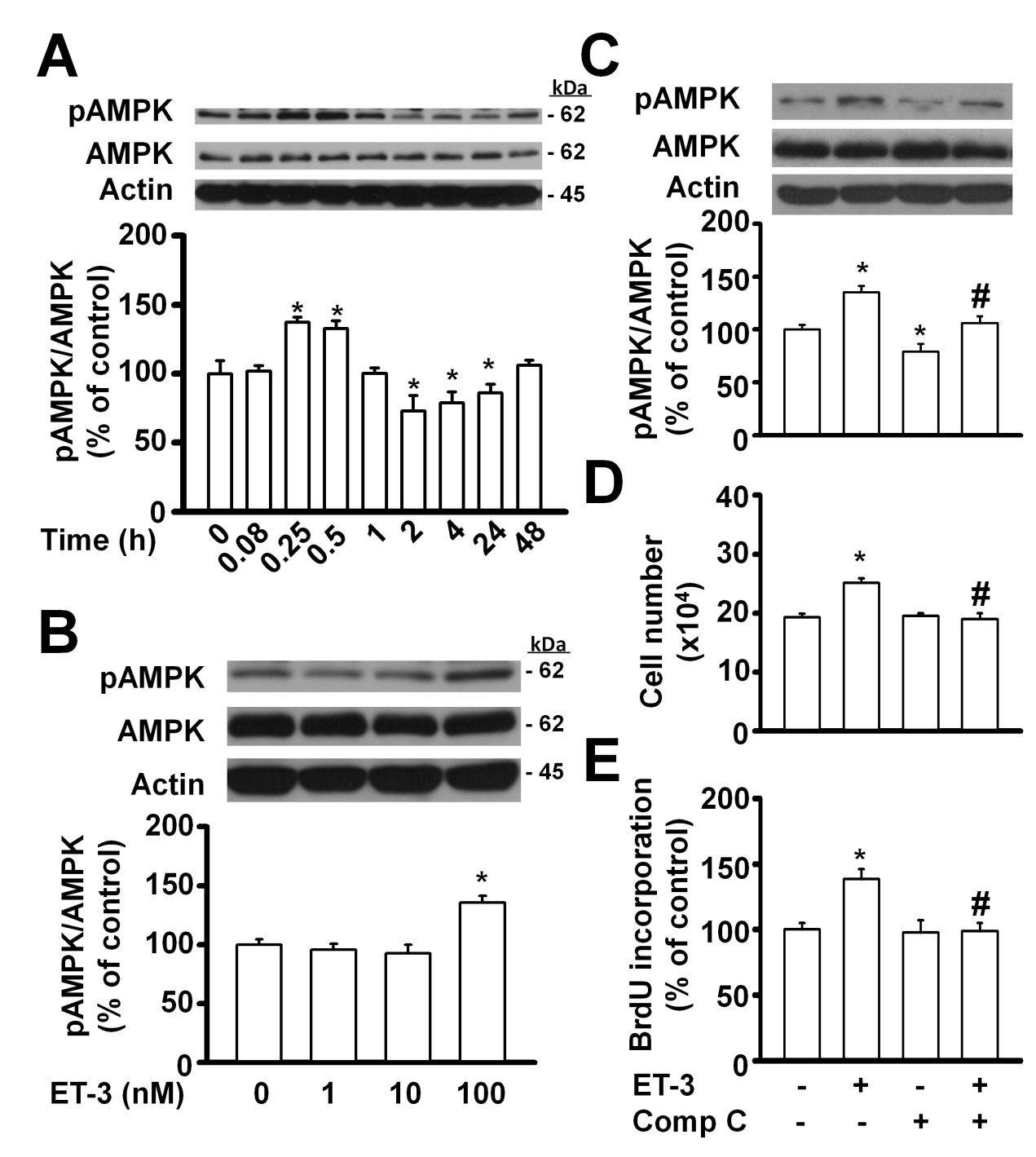


**Supplementary Figure 4.** Specific inhibitors of AMPK, such as Compound C, antagonized ET-3-induced cell growth in 3T3-L1 preadipocytes. Detection of protein expression and cell proliferation in the 3T3-L1 preadipocytes treated with compound C or ET-3: (**A**) At different time points measured by western blots and the results assessed at time 0; (**B**) At different dosages measured by western blots and the result normalized to actin; (**C**) Data were analysed by western blots and the result normalized to actin. Scale bar = 1 cm; (**D-E**) Cell proliferation measurements by trypan blue staining and BrdU incorporation. All data are expressed as the mean ± SEM deviation of three independent experiments. * *p* <0.05 vs. the controls; # *p* < 0.05, ET-3 vs. Compound C + ET-3


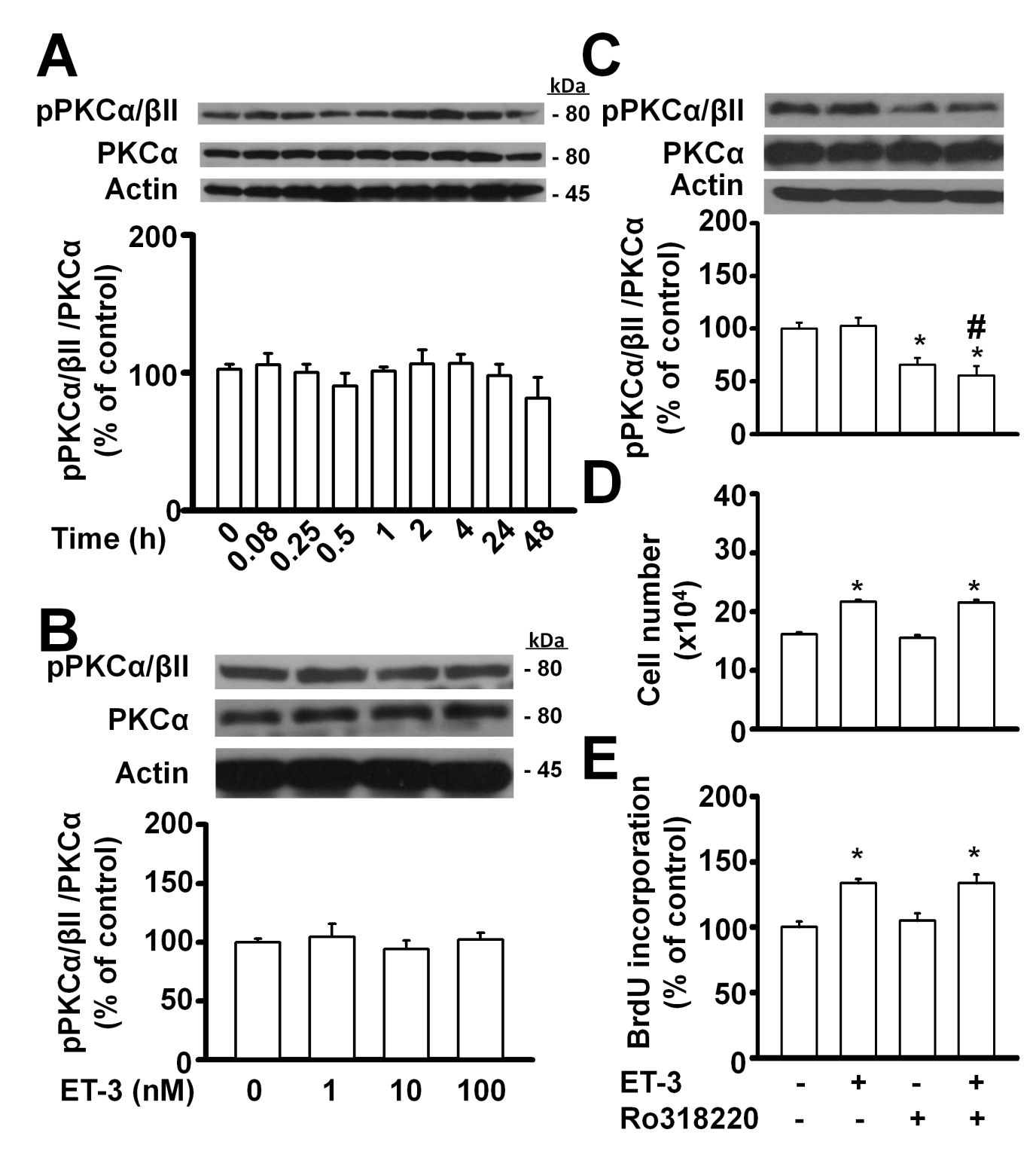


**Supplementary Figure 5.** ET-3 had no significant effect on PKC protein in 3T3-L1 preadipocytes. Detection of protein expression and cell proliferation in the 3T3-L1 preadipocytes treated with Ro318220 or ET-3: (**A**) At different time points measured by western blots and the results assessed at time 0; (**B**) At different dosages measured by western blots and the results normalized to actin; (**C**) Data were analysed by western blots and the result normalized to actin. Scale bar = 1 cm; (**D-E**) Cell proliferation measurements by trypan blue staining and BrdU incorporation. All data are expressed as the mean ± SEMof three independent experiments. * *p* <0.05 vs. the controls; # *p* < 0.05, ET-3 vs. Ro318220 + ET-3


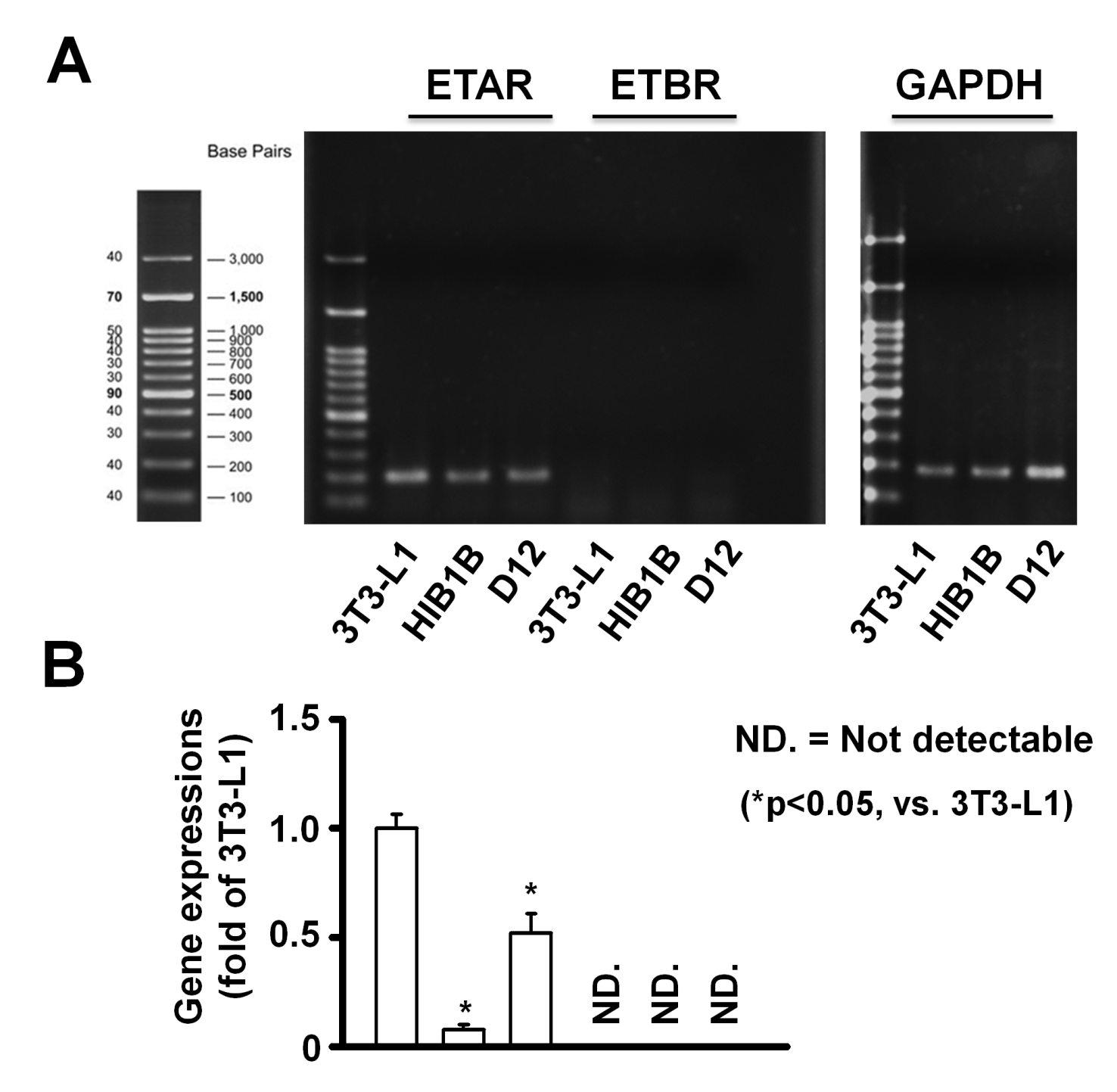


**Supplementary Figure 6.** Expression of ETAR and ETBR genes in 3T3-L1, HIB1B, and D12 cells after analysis of RT-PCR (A) and real-time PCR (B). Data were expressed as means ± SEM. *, p < 0.05, vs. 3T3-L1


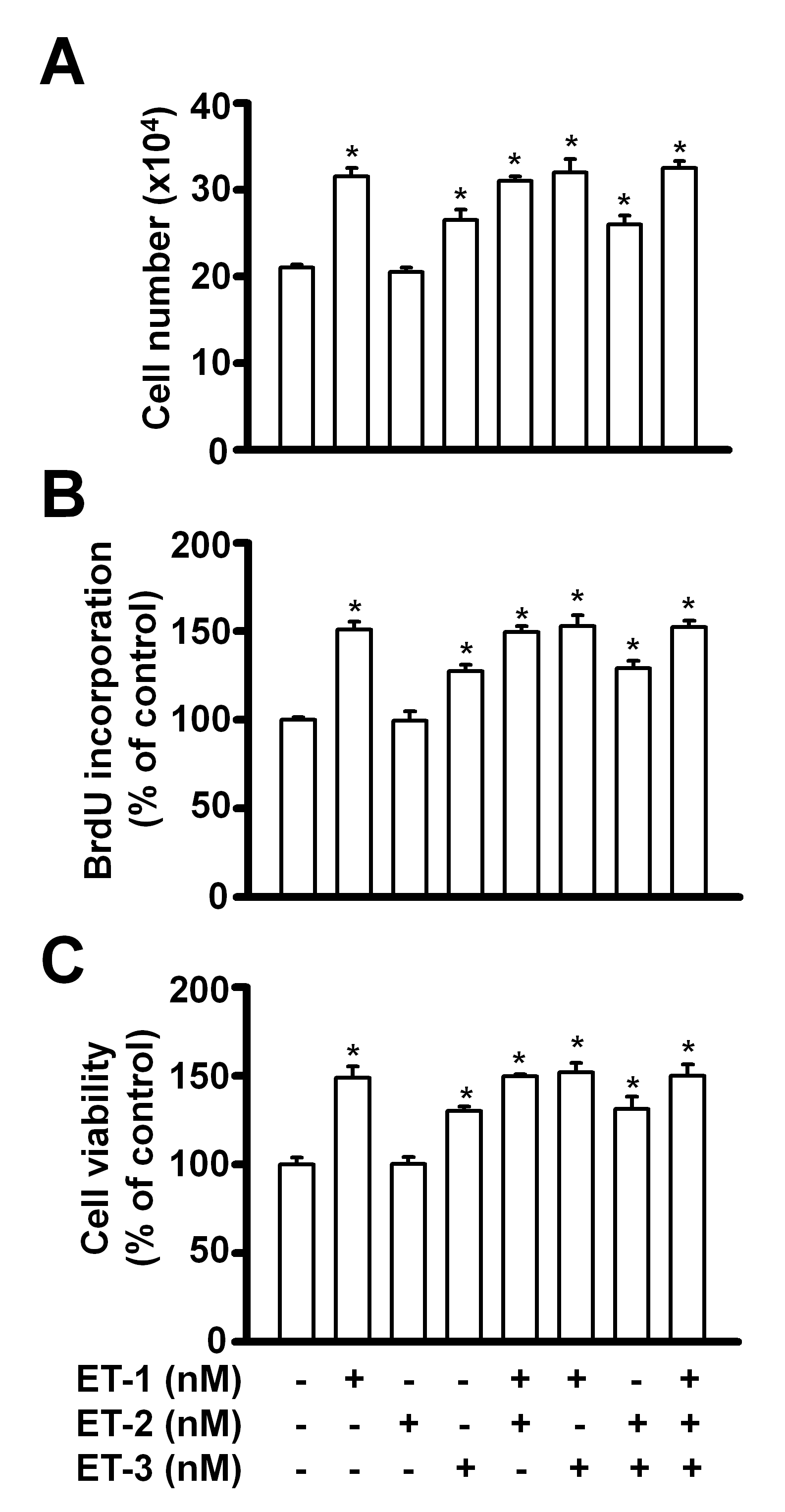


**Supplementary Figure 7.**  Differential effects of endothelin (ET)-1, ET-2, and ET-3 on the cell number (A), BrdU incorporation (B), and cell viability (C) of 3T3-L1 preadipocytes were observed. The cell number, BrdU incorporation, and cell viability were respectively detected after 48 h, 18 h, and 48 h of 100-nM ET treatments. *, P < 0.05, vs the control


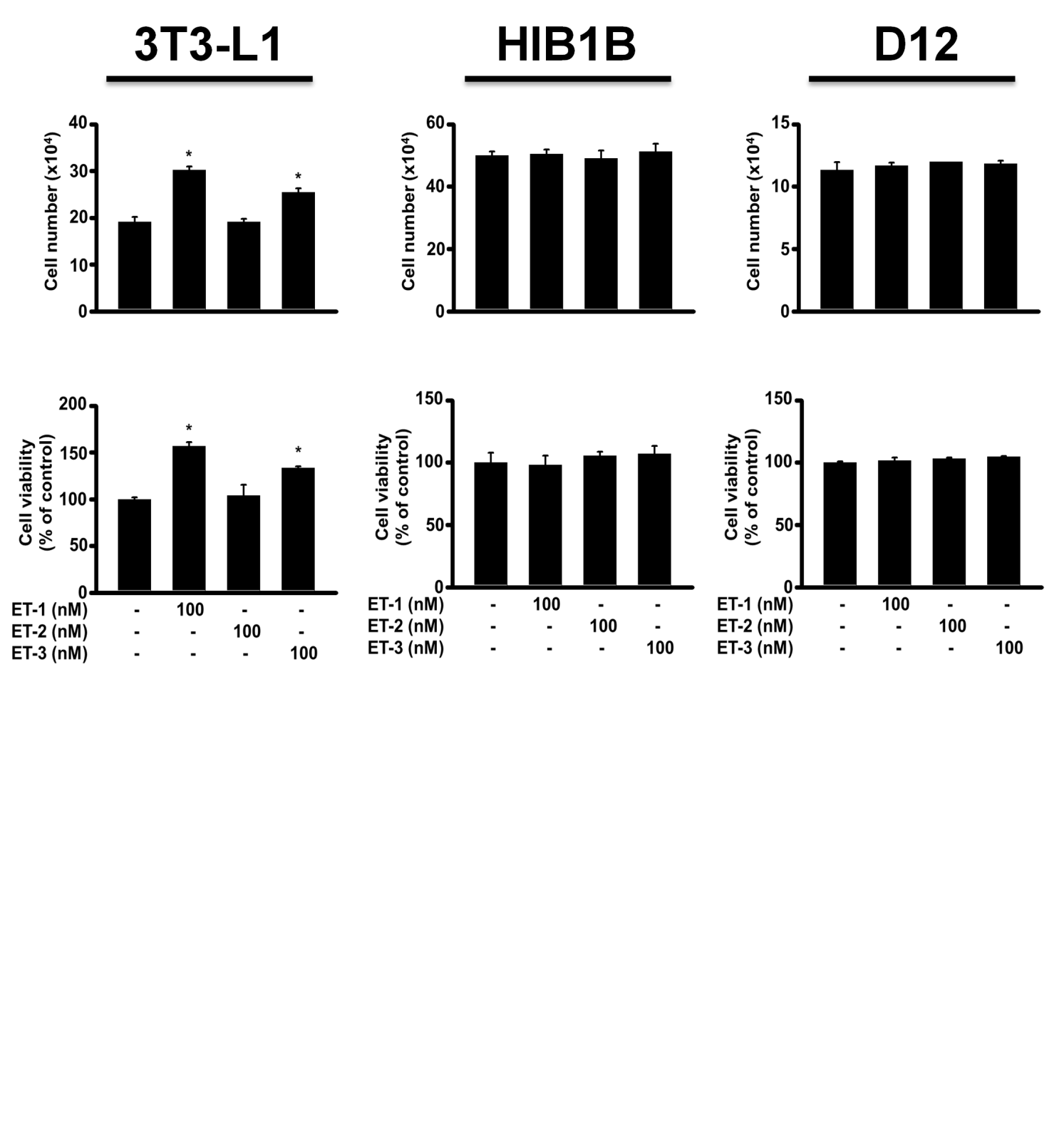


**Supplementary Figure 8.**  Differential effects of endothelin (ET)-1, ET-2, and ET-3 on the cell number and cell viability of 3T3-L1 white preadipocytes, HIB1B brown preadipocytes, and D12 beige preadipocytes were observed after 48 h of 100-nM ET treatment. *, P < 0.05, vs the control


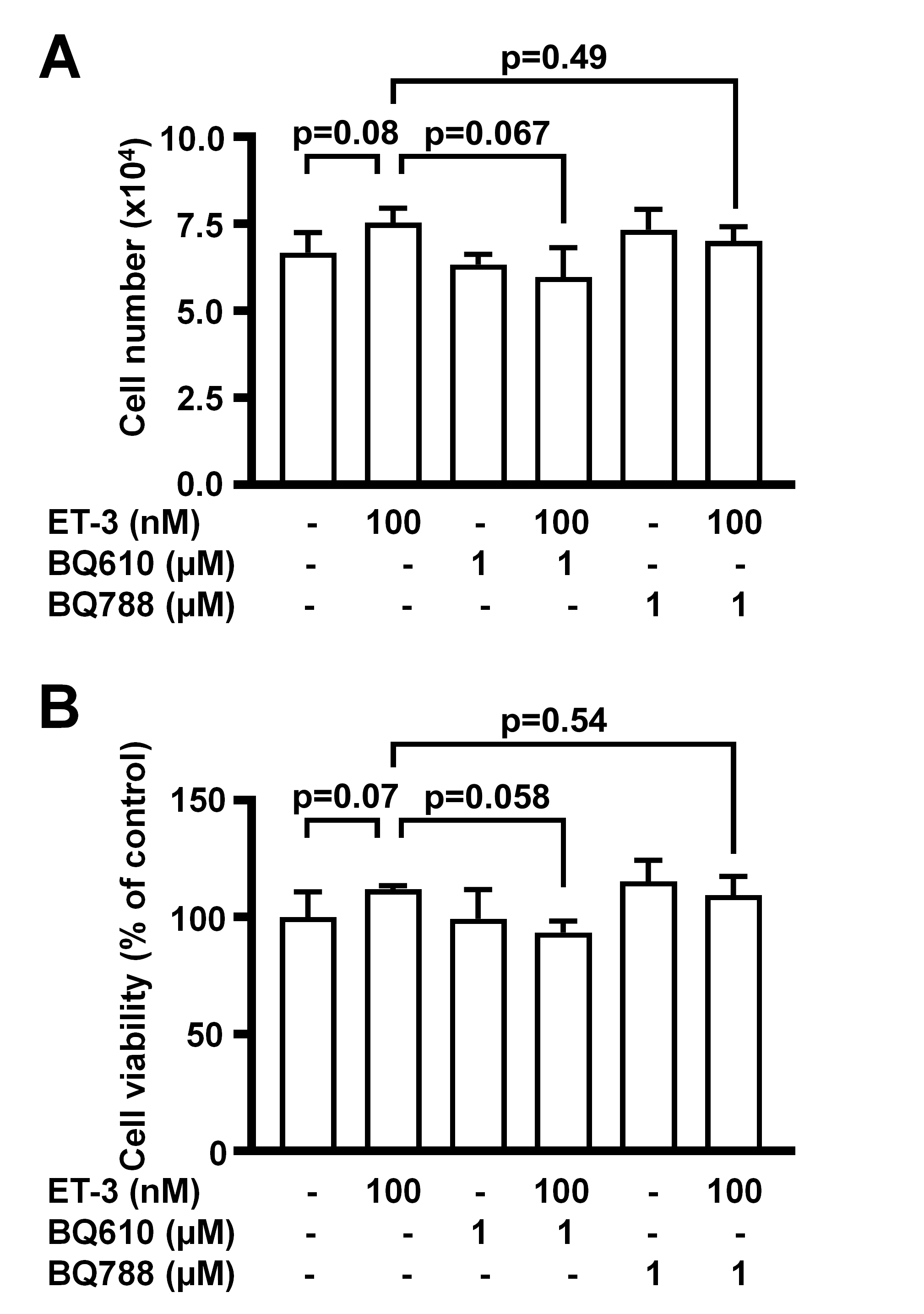


**Supplementary Figure 9.**  Endothelin (ET)-3 at 100 nM tended to stimulate the growth of primary preadipocytes derived from the stromal fraction of mouse epididymal adipose tissues, as indicated by increased cell number (**A**; P = 0.08) and increased cell viability (**B**; P = 0.07). The effect was blocked after 1 μM of BQ610 treatment.
