# Supplementary material for: Investigation of the Molecular Mechanisms by Which Endothelin-3 Stimulates Preadipocyte Growth
Source: Front Endocrinol (Lausanne). 2021 May 21;12:661828. doi: 10.3389/fendo.2021.661828 (PMC8176213; doi:10.3389/fendo.2021.661828)
Supplement: Supplementary file 2 [file Table_1.docx]

| **Supplementary table 1. Antibodies used in the experiments.** | | | | |
| --- | --- | --- | --- | --- |
| **Antibodies** | **Vendor, Cat Num** | **Species raised in; monoclonal or polyclonal** | **Dilution used** |  |
| **Primary Antibody** |  |  |  |  |
| Actin | Cell Signaling Technology, 12620 | Rabbit; monoclonal | 1:6000 |  |
| Akt1/2/3 (H-136) | Santa Cruz Biotechnology, sc-8312 | Rabbit; polyclonal | 1:1000 |  |
| AMPKα Antibody | Cell Signaling Technology, 2793 | Mouse; monoclonal | 1:1000 |  |
| c-Jun (60A8) rabbit mAb | Cell Signaling Technology, 9165 | Rabbit; monoclonal | 1:1000 |  |
| ERK1 (C16) | Santa Cruz Biotechnology, sc-93 | Rabbit; polyclonal | 1:1000 |  |
| JNK1 (F3) | Santa Cruz Biotechnology, sc-1648 | Mouse; monoclonal | 1:1000 |  |
| p38 MAPK Antibody | Cell Signaling Technology, 9212 | Rabbit; polyclonal | 1:1000 |  |
| PKCα Antibody | Cell Signaling Technology, 2056 | Rabbit; polyclonal | 1:1000 |  |
| Stat3 Mouse mAb | Cell Signaling Technology, 9139 | Mouse; monoclonal | 1:1000 |  |
| Phospho-Akt (Ser473) Antibody | Cell Signaling Technology, 4058 | Rabbit; monoclonal | 1:1000 |  |
| Phospho-AMPK (Thr172) rabbit mAb | Cell Signaling Technology, 2535 | Rabbit; monoclonal | 1:1000 |  |
| phospho-c-Jun (KM-1) | Santa Cruz Biotechnology,sc-822 | Mouse; monoclonal | 1:1000 |  |
| phospho-JNK (G7) | Santa Cruz Biotechnology,sc-6254 | Mouse; monoclonal | 1:1000 |  |
| Phospho-p44/42 MAPK(Erk1/2)(Thr202/Tyr204) Antibody | Cell Signaling Technology, 9101 | Rabbit; polyclonal | 1:1000 |  |
| Phospho-Stat3 (Tyr705) rabbit mAb | Cell Signaling Technology, 9145 | Rabbit; monoclonal | 1:1000 |  |
| Phospho-p38 MAP Kinase (Thr180/Tyr182) Antibody | Cell Signaling Technology, 9211 | Rabbit; polyclonal | 1:1000 |  |
| Phospho-PKCα/β II (Thr638/641) Antibody | Cell Signaling Technology, 9375 | Rabbit; polyclonal | 1:1000 |  |
| **Secondary Antibody** |  |  |  |  |
| Anti-mouse IgG, HRP-linked Antibody | Cell Signaling Technology, 7076 | Horse; polyclonal | 1:2000 |  |
| Anti-rabbit IgG, HRP-linked Antibody | Cell Signaling Technology, 7074 | Goat; polyclonal | 1:2000 |  |
